# Myiasis in domestic cats: a global review

**DOI:** 10.1186/s13071-019-3618-1

**Published:** 2019-07-29

**Authors:** Marco Pezzi, Teresa Bonacci, Marilena Leis, Elisabetta Mamolini, Maria Gabriella Marchetti, Stjepan Krčmar, Milvia Chicca, Carlo Nicola Francesco Del Zingaro, Michel J. Faucheux, Chiara Scapoli

**Affiliations:** 1 Department of Life Sciences and Biotechnology, University of Ferrara, Via Luigi Borsari 46, 44121 Ferrara, Italy; 2 Department of Biology, Ecology and Earth Science, University of Calabria, Via P. Bucci, Arcavacata di Rende, 87036 Cosenza, Italy; 3 Department of Biology, Josip Juraj Strossmayer University of Osijek, Cara Hadrijana 8/A, HR-31000 Osijek, Croatia; 4 Veterinary Clinic, Via Reale 124, Alfonsine, 48011 Ravenna, Italy; 5 Laboratoire d’Endocrinologie des Insectes Sociaux, Université de Nantes, 2 rue de la Houssinière, B. P. 92208, 44322 Nantes Cedex 03, France

**Keywords:** Domestic cat, Calliphoridae, Literature review, Muscidae, Myiasis, Oestridae, Sarcophagidae

## Abstract

Myiasis is an infestation caused by larvae of Diptera in humans and other vertebrates. In domestic cats, *Felis silvestris catus* L. (Carnivora: Felidae), four dipteran families have been reported as agents of obligatory and facultative myiasis: Oestridae, Calliphoridae, Sarcophagidae and Muscidae. Among agents of obligatory myiasis, the most frequent genus is *Cuterebra* Clark (Oestridae) and the most frequent species is *Cochliomyia hominivorax* (Coquerel) (Calliphoridae). Among the agents of facultative myiasis, the most frequent species is *Lucilia sericata* (Meigen) (Calliphoridae). A survey of myiasis in cats reported in literature shows that the cases are distributed worldwide and linked to the geographical range of the dipteran species. Factors favouring the occurrence of myiasis in cats are prowling in infested areas, poor hygiene conditions due to diseases and/or neglect, and wounds inflicted during territorial or reproductive competition. The aim of the review is to provide an extended survey of literature on myiasis in cats, as general information and possible development of guidelines for veterinarians, entomologists and other researchers interested in the field.

## Background

Diseases caused by insect larvae invading the body of animals were initially named “scolechiasis” [1] without any distinction of insect species, from the historical term “scolex” indicating a larva. In 1840, Reverend Frederick W. Hope introduced a new name for this type of disease based on the insect order of larvae infesting the human body: “myiasis” for Diptera, “chantariasis” for Coleoptera and “scholechiasis” for Lepidoptera [2]. Later, Professor Walter S. Patton extended the term “myiasis”, defining it as “the condition or conditions, resulting from the invasion of tissues and organs of man and other animals by all stages of the Diptera” [3]. According to this statement, myiasis is not only caused by larvae but also by all dipteran life stages. In his monography “The flies that cause myiasis in man”, Professor Maurice T. James criticized the definition of Patton as “unnecessary” and “confusing”, “since … it is the larva that is the active stage in relation to myiasis” [4]. The current and widely accepted definition of myiasis is that by Professor Fritz K. Zumpt, formulated “as the infestation of live human and vertebrate animals with dipterous larvae, which, at least for a certain period, feed on the host’s dead or living tissue, liquid body-substances, or ingested food”. The key criterion to define myiasis is therefore the fact that the dipteran larva must complete its normal development or a part of it in a vertebrate body [5].

Myiasis can be classified based on host-parasite relationships, that is whether the dipteran species cause obligatory or facultative myiasis. The species causing obligatory myiasis require a living host for their development, while those causing facultative only occasionally lay eggs or larvae on living hosts and usually develop on decaying matter. Facultative myiasis can be classified as primary, secondary and tertiary, based on the ability of the species to initiate the myiasis (primary) or to superimpose to pre-existing myiasis (secondary and tertiary). Myiasis can also be classified based on anatomical localization of the larva (or larvae) in the host, either external or internal: auricular, cutaneous, gastrointestinal, ophthalmic, oral and urogenital [6–8]. Besides these types, myiasis involving structures of the nervous system will be considered as “neurological” in this review and those involving structures of the respiratory system will be considered as “respiratory”.

This review summarizes the worldwide reported cases of myiasis in domestic cats (hereafter cats), *Felis silvestris catus* L. (Carnivora: Felidae), the dipteran species involved and their ecological and biological features. The parasitological, epidemiological and risk factors will also be examined in detail, in order to provide information and suitable guidelines for veterinarians, entomologists and other researchers interested in the field.

## Bibliographic methods

More than 7000 published references were examined for this study. The initial set of publications on myiasis in cats were obtained by PubMed indexed literature and the search was extended through web engines. The search had no time and language limits. For each publication obtained *via* web or interlibrary services or by direct contacts with the authors, the reference list was checked for further cases of myiasis in cats. Publications not specifically reporting myiasis on cats in their titles were also investigated, examining the abstracts or the entire publication for useful information. All publications reporting myiasis on cats were classified according to infesting taxon, type of myiasis, number of cases, sex, age and country. The literature data were then described according to the Diptera family and to the genera or species agents of obligatory and facultative myiasis.

## Literature data

The first cases of myiasis in cats reported in scientific journals appeared around the end of the 19th century in the USA [9, 10]. A case of cutaneous myiasis in a foot of a cat from Texas, due to a fighting wound in which numerous “screw-worms” developed, was reported in a letter by Professor G. W. Curtis to the distinguished entomologist, Professor Herbert Osborn, dated 15 December 1888; the agent involved was presumably *Cochliomyia* sp. (Diptera: Calliphoridae) [10].

A case of myiasis in a cat from Iowa by *Cuterebra* sp. was later reported by Osborn in 1892 [11], but the original work could not be retrieved. A case of “warble” fly on the belly of a cat from North Carolina was described in the extracts from correspondence of the journal “Insect Life”; the editors suggested it could be due to a species of the genus *Cuterebra* [9]. Another two cases of myiasis (“bots”) were reported in the same journal in the notes from correspondents, the first one in the eye and back of a cat in New York State and the second one in the neck of a kitten in Missouri; in both cases no identification of the agents was provided [9]. From 1894 onwards, the cases of myiasis in cats were described as caused by species of the families Oestridae, Calliphoridae, Sarcophagidae and Muscidae (Tables 1, 2, 3, 4, 5 and 6). The cases of myiasis are therefore reported according to the dipteran family involved.

**Table 1:** Genera and species of Diptera involved in myiasis in cats

Family	Genus/Species	Obligatory/Facultative
Oestridae	*Cuterebra* sp.	O
	*Cuterebra horripilum*	O
	*Dermatobia hominis*	O
	*Oestrus ovis*	O
Calliphoridae	*Cochliomyia hominivorax*	O
	*Chrysomya bezziana*	O
	*Cordylobia anthropophaga*	O
	*Lucilia sericata*	F
	*Calliphora vicina*	F
	*Phormia regina*	F
	*Lucilia* sp.	F
	*Lucilia eximia*	F
	*Lucilia ampullacea*	F
	*Lucilia caesar*	F
	*Lucilia coeruleiviridis*	F
Sarcophagidae	*Sarcophaga* sp.	F
	*Sarcophaga tibialis*	F
	*Sarcophaga argyrostoma*	F
	*Wohlfahrtia* sp.	O
	*Wohlfahrtia vigil*	O
	*Wohlfahrtia magnifica*	O
Muscidae	*Musca domestica*	F

*Abbreviations*: O, obligatory; F, facultative

**Table 2:** Cases of myiasis in cats caused by taxa of the family Oestridae

Taxon	Type of myiasis	No. of cases	Sex	Age	Country	References
*Cuterebra* sp.	neu	1	unk	unk	USA	[62]
	neu	1	♂	A	USA	[54]
	neu	1	♀	K	USA	[64]
	neu	2	♀	A	USA	[43]
	neu	3	2♀; 1♂	1K; 2A	USA	[65]
	neu	11	6♀; 5♂	A	USA	[44]
	neu	9	5♀; 4♂	7A; 2unk	USA	[45]
	neu	1	♀	A	USA	[47]
	neu	1	♀	A	USA	[50]
	ophth	1	unk	8w to 2y	USA	[42]
	ophth	1	♀	A	USA	[56]
	ophth	1	♀	A	USA	[46]
	ophth	1	♀	A	USA	[48]
	ophth	1	♀	A	USA	[73]
	oral	1	unk	unk	USA	[63]
	oral	1	unk	unk	USA	[51]
	resp	1	unk	unk	USA	[11]
	resp	1	♀	K	USA	[61]
	resp	2	♂	1A; 1K	USA	[57]
	resp	1	♂	A	USA	[59]
	resp	2	1♀; 1♂	A	USA	[60]
	resp	1	♂	A	USA	[74]
	resp	1	♂	A	Canada	[66]
	resp	1	unk	unk	USA	[51]
	cut	1	unk	K	USA	[39]
	cut	2	unk	unk	USA	[11]
	cut	1	unk	unk	USA	[41]
	cut	6	unk	8w to 2y	USA	[42]
	cut	20	unk	unk	USA	[51]
	cut	1	unk	K	USA	[49]
	aur	1	♂	A	USA	[58]
	neu-cut	1	♀	K	USA	[65]
	neu-cut	1	♀	A	USA	[45]
	unk	1	unk	unk	USA	[40]
*C. horripilum*	cut	2	unk	K	USA	[20]
*Dermatobia hominis*	cut	1	unk	A	Brazil	[78]
	cut	1	unk	unk	Venezuela	[79]
	cut	1	♀	A	Brazil	[80]
*Oestrus ovis*	resp	1	♂	A	Australia	[81]
Cuterebrinae	unk	2	unk	1K; 1A	USA	[19]
Oestridae	cut	1	unk	K	USA	[169]
	cut	1	unk	unk	Canada	[170]

*Abbreviations*: neu, neurological; ophth, ophthalmic; cut, cutaneous; resp, respiratory tract; unk, unknown; A, adult; K, kitten; w, weeks; y, years

**Table 3:** Cases of myiasis in cats caused by taxa of the family Calliphoridae

Taxon	Type of myiasis	No. of cases	Sex	Age	Country	References
*Cochliomyia* sp.	cut	1	unk	A	USA	[10]
*Cochliomyia hominivorax*	ophth	1	♀	A	Brazil	[88]
	oral	1	♂	A	Brazil	[88]
	cut	2	unk	unk	USA	[92]
	cut	3	unk	unk	USA	[93]
	cut	2	unk	unk	Trinidad and Tobago	[94]
	cut	2	unk	unk	Jamaica	[95]
	cut	1	unk	unk	Argentina	[171]
	cut	9	unk	unk	Argentina	[96]
	cut	10	1♀; 9♂	A	Brazil	[88]
	cut	5	♂	A	Brazil	[90]
	cut-oral-resp	1	♂	A	Brazil	[89]
	unk	unk	unk	unk	USA	[172]
	unk	2	unk	unk	Curaçao	[173]
	unk	unk	unk	unk	Brazil	[86]
	unk	>152	unk	unk	Brazil	[87]
	unk	1	unk	unk	Brazil	[91]
	unk	2	unk	unk	USA	[85]
*Chrysomya bezziana*	cut	21	3♀; 18♂	1K; 20A	Malaysia	[100]
	unk	unk	unk	unk	Papua New Guinea	[97]
	unk	1	unk	unk	Papua New Guinea	[98]
	unk	4	unk	unk	Sri Lanka	[99]
*Lucilia sericata*	ophth	1	unk	A	Turkey	[120]
	cut	1	unk	unk	Germany	[111]
	cut	1	♂	unk	Argentina	[125]
	cut	10	unk	unk	Argentina	[96]
	cut	1	unk	unk	Israel	[119]
	cut	2	1unk; 1♀	unk	Malta	[115]
	cut	2	♀	A	Italy	[117]
	cut	1	♂	A	Turkey	[121]
	cut	1	♀	unk	Turkey	[123]
	urog	1	♀	unk	USA	[124]
	urog	1	♀	A	Turkey	[118]
	urog	1	♀	A	Turkey	[122]
	gastr	1	unk	unk	Italy	[108]
	gastr	2	unk	unk	Israel	[119]
	unk	unk	unk	unk	Austria	[113]
	cut-gastr-urog	4	unk	unk	Austria	[112]
*Calliphora vicina*	cut	3	♂	A	Spain	[134]
	aur	1	unk	K	Italy	[117]
*Phormia regina*	cut-aur-gastr	3	unk	K	Turkey	[144]
*Lucilia eximia*	cut	1	♀	K	Brazil	[146]
*Lucilia caesar*	unk	unk	unk	unk	Austria	[113]
*Lucilia coeruleiviridis*	unk	1	unk	K	USA	[154]
*Lucilia* sp.	unk	unk	unk	unk	Canada	[174]

*Abbreviations*: urog, urogenital; gastr, gastrointestinal; aur, auricular; ophth, ophthalmic; cut, cutaneous; resp, respiratory tract; unk, unknown; A, adult; K, kitten

**Table 4:** Cases of myiasis in cats caused by association of species of Diptera

Species involved	Type of myiasis	No. of cases	Sex	Age	Country	Reference
*Lucilia sericata* and *Lucilia ampullacea*	unk	1	unk	unk	Austria	[114]
*L. sericata* and *Sarcophaga tibialis*	cut	1	unk	A	Italy	[116]
*L. sericata* and *Calliphora vicina*	ophth	1	unk	K	Italy	[117]

*Abbreviations*: ophth, ophthalmic; cut, cutaneous; unk, unknown; A, adult; K, kitten

**Table 5:** Cases of myiasis in cats caused by taxa of the families Sarcophagidae and Muscidae

Family	Taxon	Type of myiasis	No. of cases	Sex	Age	Country	References
Sarcophagidae	*Sarcophaga* sp.	unk	unk	unk	K	Canada	[165]
	*S. argyrostoma*	cut	1	♂	A	Italy	[117]
	*Wohlfahrtia* sp.	unk	unk	unk	K	Canada	[165]
		unk	1	unk	unk	USA	[67]
	*W. magnifica*	cut	1	unk	unk	Israel	[119]
		oral	1	unk	A	Italy	[161]
	*W. vigil*	cut	4	unk	unk	Canada	[162]
		cut	2	unk	K	Canada	[163]
		cut	1	unk	K	Canada	[164]
		cut	3	unk	unk	USA	J. T. Alfred (pers. comm., 2017)
		unk	1	unk	unk	USA	[67]
Muscidae	*Musca domestica*	resp	1	unk	A	USA	[168]

*Abbreviations*: cut, cutaneous; resp, respiratory tract; unk, unknown; A, adult; K, kitten

**Table 6:** Cases of myiasis in cats caused by undetermined taxa of Diptera

Type of myiasis	No. of cases	Sex	Age	Country	References
ophth	1	♂	A	USA	[175]
ophth	2	♀	A	USA	[176]
ophth	2	1♀; 1♂	1A; 1 unk	USA	[177]
cut	2	unk	1K; 1 unk	USA	[9]
cut	2	unk	unk	USA	[92]
cut-ophth	1	unk	A	USA	[9]

*Abbreviations*: ophth, ophthalmic; cut, cutaneous; unk, unknown; A, adult; K, kitten

### Oestridae

The Oestridae is a large dipteran family in which all species are obligate parasites of wild and domestic animals and of humans. It is divided into four subfamilies: Cuterebrinae (New World skin bot flies), Oestrinae (nose bot flies), Gasterophilinae (stomach bot flies) and Hypodermatinae (Old World skin bot flies) [6, 7]. The cases of myiasis in cats reported as caused by species of the Oestridae are indicated in Table 2, almost all belonging to the Cuterebrinae and Oestrinae. Within the subfamily Cuterebrinae, species of the genus *Cuterebra* and *Dermatobia hominis* Linnaeus Jr. in Pallas, a species of the monotypic *Dermatobia*, both from the New World [6, 12] have been reported as agents of myiasis in cats.

The species of genus *Cuterebra* exhibit a marked host specifity: some species preferentially attack rodents such as voles, *Microtus* spp., woodrats, *Neotoma* spp., and deer mice, *Peromyscus* spp. (Cricetidae), and lagomorphs such as hares, *Lepus* spp., and cottontail rabbits, *Sylvilagus* spp. (Leporidae) [7]. They may also develop in atypical rodent hosts, such as black rats, *Rattus rattus* (L.) (Muridae) [13], and European rabbits, *Oryctolagus cuniculus* (L.) (Leporidae) [14]. Besides cats (Table 2), other atypical mammalian hosts may be affected, among which are red kangaroos, *Macropus rufus* Desmarest and Bennett’s wallabies, *Macropus rufogriseus fruticus* (Ogilby) (Diprotodontia: Macropodidae) [15], cattle, *Bos taurus* L. [16] and Günther’s dik-diks, *Madoqua guentheri* Thomas [15] (Artiodactyla: Bovidae), domestic pigs, *Sus scrofa domesticus* Erxleben (Artiodactyla: Suidae) [16], horses, *Equus ferus caballus* L. [17] and mules, *Equus asinus* L. × *E. ferus caballus* [16] (Perissodactyla: Equidae), domestic dogs, *Canis lupus familiaris* L. (Carnivora: Canidae) [18–25], snow leopards, *Panthera uncia* (Schreber) (Carnivora: Felidae) [26], raccoons, *Procyon lotor* L. (Carnivora: Procyonidae) [27] and primates such as ring-tailed lemurs, *Lemur catta* L. (Lemuridae) [28] and humans [29–36]. Myiasis caused by species of the genus *Cuterebra* has also been reported in non-mammalian species such as reptiles [37] and birds [38].

In cats, *Cuterebra* spp. have been reported to cause neurological, ophthalmic, oral, respiratory and furuncular (cutaneous) myiasis (Table 2). Infestations were detected from June to September [39–51]. Emerging of adults occurred from late spring, followed by mating, oviposition and egg development [52]. The common ways to enter the host are natural body openings or wounds [53]. Species have been clearly determined only in one case, probably because the authors typically focused on clinical symptoms, treatments and outcomes of infestations: *Cuterebra horripilum* Clark was reported in a case involving two kittens in the USA [20]. In another case involving an adult male, the author mentioned the species as *Cuterebra americana* (Fabricius) or *C. horripilum* [54]. In another recent case of myiasis in a kitten the author reported that it was “most likely” *Cuterebra abdominalis* Swenk [49]. The recent availability of molecular techniques such as DNA barcoding may provide a precise identification of the species, as for other Diptera responsible for myiasis [55]. All cases of myiasis in cats by *Cuterebra* spp. have been reported in North America. The US states where these cases were reported and/or the cats were clinically treated are the following, according to the region. Midwest: Illinois [56], Indiana [41, 57], Iowa [11], Michigan [51, 58, 59], Missouri [60], Ohio [48] and Wisconsin [46, 49]; Northwest: Connecticut [39, 40], Massachusetts [61] and New York [44, 45, 47, 62]; Southwest: North Carolina [63]; South: Alabama [64, 65], Georgia [43, 50, 65], Maryland [54], Tennessee [11], Texas [42] and Washington D.C. [11]. Recently, a case of respiratory tract myiasis with an infestation of pharynx caused by *Cuterebra* sp. was reported in the province of Ontario, Canada, together with two presumptive cases of neurological myiasis in which, however, no dipteran larva was found [66].

Species of the genus *Cuterebra* usually lays eggs on grasses along narrow trails or paths used by the typical host [52]; thus cats and other atypical hosts may become parasitized. While strolling, the cat sniffs the ground or brushes the contaminated area, accidentally collecting eggs or larvae [29, 50, 67]. Contacts with the parasites are more frequent in adults, while in kitten infestations are related to the daily activities of the mother, which may collect the parasites in her fur and carry them to the litter [67]. The consequences of myiasis by *Cuterebra* spp. depend on the site of infestation.

*Cuterebra* spp. larvae have been found inside the cat skin, causing “subcutaneous abscesses” [41], “lesions” [49] and “seeping wounds” [42]. The ability to use skin wounds or lacerations as a way to enter the host has been reported for *Cuterebra latifrons* Coquillett [68] and *Cuterebra fontinella* Clark [69]. Experimental infestations in rats by *Cuterebra tenebrosa* Coquillett were obtained by placing first-stage larvae on fur [70] and in humans by *Cuterebra* spp. larvae placed on intact skin [71], but these results were not confirmed for larvae of *Cuterebra lepivora* Coquillett in rabbits [72]. In cats, larvae of *Cuterebra* spp. were detected in the skin of head, neck, shoulder, thorax, abdomen and sides [11, 20, 42, 49, 51]. In all these cases, the only method to remove the parasite was mechanical extraction by surgery.

When the larva migrates to the brain, the invariable outcome is death [43, 64, 65] either natural or induced by euthanasia practiced by veterinarians [43, 45, 47, 50, 54]. The neurological signs depend on the area of the brain infested [67] and include ataxia, blindness, circling, head tilt, lethargy, seizures, hysteria and convulsion [43–45, 47, 50, 64, 65]. In some cases, aggressive behaviours such as clawing, irritability to noise and hissing often mislead veterinarians to diagnose rabies and therefore immediately euthanize the cat [43, 50]. Indeed, most cases of brain migration of *Cuterebra* spp. have been correctly diagnosed only after necroscopy and the commonly reported clinical signs before the onset of neurological symptoms are respiratory problems, probably due to larval migration in the respiratory tract [43–45, 47, 50, 64, 65].

Among the five reported cases of ophthalmomyiasis by *Cuterebra* spp. in cats, one was external [42] and the other four were internal: in three cases the larva was in the anterior chamber of the eye [46, 56, 73] and in one case in the posterior chamber [48]. When the larva was in the anterior chamber, it was found dead [46, 56, 73], presumably because of the unsuitable site of migration [73]. The dead larva may have caused an intense inflammatory reaction [56]. The larvae in the anterior chamber were removed by surgery with survival of cats in all three cases, but blindness occurred in two cases by retinal degeneration [46] and corneal edema [56]. When the larva migrated to the posterior chamber, it caused coagulation necrosis, haemorrhagic lesions to retina and optical nerve and anterior uveitis. The systemic conditions of the patient worsened so the cat had to be euthanized [48].

Other sites where larvae of *Cuterebra* spp. have been found are the oral cavity, including mandible [51], soft palate [63] and pharynx [57], and the respiratory tract, such as nasal cavity [11, 51, 61] and trachea [59, 60, 74]. When the larva infested the oral cavity, the reported clinical signs were lethargy, listlessness and anorexia [57] and when it infested the nasal cavity, the signs were sneezing and nasal discharge [61]. If the larva reached the pharynx, the signs were dyspnea and caked blood on nostrils [57], and if it reached the trachea, they were dyspnea, subcutaneous emphysema, cyanosis, stridor, coughing, sneezing, inappetence, lethargy and fever [59, 60, 74].

When the above symptoms are detected in late summer or early autumn, the veterinarian should consider the possibility of an oral or respiratory infestation by *Cuterebra* spp. [60]. In cases of oral and respiratory myiasis by *Cuterebra* spp., the only attempts to remove the infesting larvae were by surgery [57, 60, 61, 63, 74], successful in all cases except one, where the larva was extracted from the soft palate and the cat died for debilitation and serious respiratory problems [63]. A case of tracheal infestation by *Cuterebra* sp. was described as a *post-mortem* diagnosis in which death was probably due to tissue damages caused by migration and anaphylaxis [59].

Another species of the subfamily Cuterebrinae reported as an agent of obligatory furuncular myiasis in cats is *D. hominis* (Table 2), a common fly species in central and southern America [75]. The adult female exhibits a special behaviour to ensure the contact between the eggs and the host: it usually captures other zoophilic insects or arthropods and sticks her eggs to their abdomens. When the zoophilic carrier settles on the host, the larvae of *D. hominis* hatch and penetrate the host skin [7], gaining access to the dermis. Each larva forms a furuncular lesion [8]. This species is a very serious cattle pest in tropical America and is known to parasitize man, dogs and other mammals [4]. In travellers returning from tropical America it should be considered for differential diagnosis of furuncular lesions [8]. Cats are reported as possible hosts of *D. hominis* together with other domestic and wild animals [10, 76, 77], but the first documented case of myiasis by *D. hominis* in cats was reported in 1998 in Rio de Janeiro (Brazil) in a cat showing cutaneous boils on a paw and among toes [78]. Two other cases were recently reported, one in Venezuela [79] and the other in Brazil, in which the cat showed three cutaneous boils on the cranioventral region of the neck [80]. The infestation by *D. hominis* is less aggressive in comparison to that by *Cochliomyia hominivorax* (Coquerel) (Diptera: Calliphoridae), another common agent of obligatory myiasis in Brazil, and may be considered as benign, not only because the infestation is limited to the host skin without migrations inside the body, but also because of “feline self-grooming behavior” which increases the probability of removal of *D. hominis* larvae. However, if the cat is clinically debilitated and can not perform the self-grooming behaviour, infestation may occur [80].

The only species of the subfamily Oestrinae reported as an agent of myiasis in cats is *Oestrus ovis* (L.), which deposits first stage larvae in nostrils of animal and human hosts [6]. In cats, only one case of nasal infestation by *O. ovis* was described in New South Wales (Australia), with a positive outcome probably due to early diagnosis and intervention. The first-instar larva was mechanically removed by repeated washing with saline solution. The predisposing risk factor for the cat was living in a sheep-grazing area [81].

### Calliphoridae

The family Calliphoridae (“blow flies”) includes over 1000 species distributed worldwide. From a medical and veterinary point of view, many saprophagous species of this family may cause facultative myiasis [7] and thus have forensic relevance [82, 83]. A small number of Calliphoridae are characterized by obligatory myiasis [7]. Those reported in cats are *Co. hominivorax*, *Chrysomya bezziana* Villeneuve and *Cordylobia anthropophaga* (Blanchard) (Table 3).

The first two species, respectively called “New World screw-worm” and “Old World screw-worm”, are economically relevant and occupy the same ecological niche in their respective ranges [6, 7]. All warm-blooded animals, either domestic and wild, and also humans may become hosts for these two species [84], whose females are attracted by open wounds or by mucosae of natural body openings [6]. The species *Co. hominivorax* was originally distributed from southern USA to South America [84]. From 1952 to 1982, campaigns of dispersal of sterile flies succeeded to eradicate the parasite from the USA, Mexico and several Caribbean islands. In 1987 an autochthonous spreading of *Co. hominivorax* was reported in Libya, probably originating from livestock imported from Central America. In this outbreak, sheep, goats, cattle, camels and more than 200 humans were infested, but in 1991 eradication was achieved by an international campaign of sterile fly release [84]. In 2016 an outbreak of *Co. hominivorax* occurred in the Florida Keys, with confirmed cases involving ten Key deers, *Odocoileus virginianus clavium* Barbour & G. M. Allen (Artiodactyla: Cervidae), one raccoon, three domestic dogs, one pet pig and two cats. The outbreak was ended in 2017 by sterile fly release [85]. In cats, *Co. hominivorax* has been historically detected in the USA, Curaçao, Jamaica, Trinidad and Tobago, Brazil and Argentina (Table 3). The country with the highest number of reported cases is Brazil, with reports from Rio de Janeiro [86–90] and the Federal District [91]. In Rio de Janeiro, this infestation has been reported as “quite common” in cats [90], infesting wounds [88–90, 92–96] and ears, oral and nasal cavities [88, 89]. Concerning wounds, infestation of *Co. hominivorax* have been reported in cats with surgery wounds due to castration [95] or cryosurgery [90], or with traumatic wounds due to bites of haematophagous bats [94], and territorial or reproductive fights among males [88, 90].

Infestations by *Co. hominivorax* have also been reported in the umbilical region of kittens [94]. Myiasis by *Co. hominivorax* may be fatal, especially in stray cats for which diagnosis and treatment are difficult due to feral behaviour [88, 90].

The area of distribution of the Old World screw-worm, *Ch. bezziana*, is Southeast Asia, New Guinea and Africa [7]. Contrary to its American counterpart, the number of cases of myiasis in cats reported for this species are few, located in Papua New Guinea [97, 98], Sri Lanka [99] and Malaysia [100]. Cats have been also reported as possible hosts for *Ch. bezziana* in the Sultanate of Oman [101]. In the Sri Lanka survey, among 299 cases of myiasis in domestic dogs and cats by *Ch. bezziana*, four concerned cats [99]. The authors did not advance any hypothesis about the high prevalence of myiasis in dogs in comparison to cats, an interesting point that would deserve further investigation.

The “tumbu fly”, *Cor. anthropophaga*, is an agent of obligatory cutaneous (furuncular) myiasis, widely distributed in the sub-Saharan region [6], although at least one autochtonous case has been recently reported in Saudi Arabia [102]. This species, with rodents as natural hosts, has secondarily adapted to other wild and domestic animals and also to humans [7]. Cats have been historically mentioned as hosts of the tumbu fly [5, 103] but according to scientific literature there are no specific reports of myiasis caused by this species in cats.

Species of the Calliphoridae reported as agents of facultative myiasis in cats are *Lucilia sericata* (Meigen), *Calliphora vicina* Robineau-Desvoidy, *Phormia regina* Meigen, *Lucilia caesar* (L.), *Lucilia eximia* (Wiedemann), *Lucilia ampullacea* Villeneuve and *Lucilia coeruleiviridis* Macquart (Tables 3, 4).

*Lucilia sericata* has a nearly cosmopolitan distribution and is a common necrophagous fly in temperate regions of the northern hemisphere [83]. Together with *Ca. vicina* and *L. illustris*, this species is characteristic for urban and suburban areas in Europe [104] and in northern Europe is the primary agent of cutaneous myiasis [105]. Adults thrive on carrions in bright sunshine [106] but the female searches shaded areas of the dead body to lay eggs [83]. Larvae generally feed on dead tissues but are also attracted by wounds and lesions [5], thus they are used in a kind of biotherapy known as “maggot therapy” [107]. They are well-known agents of facultative myiasis in domestic mammals and humans [5, 108–110]. The reported cases of myiasis caused by *L. sericata* in cats occurred in southern and central Europe [108, 111–117], the Middle East [118–123], North America [124] and South America [96, 125]. This species has been found in skin lesions [96, 111, 112, 115–117, 119, 121, 123, 125] caused by traumas such as falls [115], dog bites [116] and car accidents [117], decubitus ulcers and fur soiled by urine and faeces due to neglected conditions [115], old age [117] or postpartum lesions [123]. *Lucilia sericata* has been reported as agent of urogenital myiasis in three female cats, the first two bearing dead fetuses [118, 122] and the third one with postpartum problems [124]. In males, myiasis of prepuces contaminated by urine and smegma have also been described [112]. Gastrointestinal myiasis by *L. sericata* have been reported as localized in the rectal region [108] and around and inside the anal region [119]. Myiasis in the perineal region associated with diarrhea due to feline infectious peritonitis have also been reported [112]. Cases of ophtalmic myiasis by *L. sericata* have been described in a stray cat [120] and in an abandoned kitten, in association with *Ca. vicina* [117]. A case of myiasis in a cat by *L. sericata* in association with *Lucilia ampullacea* Villeneuve (Diptera: Calliphoridae) was reported in Austria [114]. Four cases of myiasis by *L. sericata* have been reported in Italy: the first three were traumatic and one of them was in association with *Sarcophaga tibialis* Macquart [116, 117]; the fourth one was ophthalmic and in association with *Ca. vicina* [117].

*Calliphora vicina*, common in the northern hemisphere [5], is a polyphagous synanthropic species [106, 126] with forensic relevance [127]. When causing myiasis, the species is characterized by a remarkable voracity and by the tendency to invade deep tissues [128]. The species has been reported as agent of myiasis in humans [109] and other mammals recently including the common noctule, *Nyctalus noctula* Schreber (Chiroptera: Vespertilionidae) and the crested porcupine, *Hystrix cristata* L. (Rodentia: Hystricidae) [129–131], and in non-mammal species such as the Hermann’s turtle, *Testudo hermanni* Gmelin (Testudines: Testudinidae) [132] and the Eastern imperial eagle, *Aquila heliaca* Savigny (Accipitriformes: Accipitridae) [133]. This species has been reported in Spain as an agent of cutaneous myiasis in three neutered cats, obese for excessive and inappropriate food [134]. Obesity prevented the cats from normal grooming, so they had painful, putrid-smelling ulcers on thighs and tails. The accumulation of anal sac secretions in the perineal region not only attracted females of *Ca. vicina* but also provided a suitable substrate for egg laying [134]. Two other cases of myiasis by *Ca. vicina* were recently reported in orphaned kitten: the first, auricular, involved a newborn still bound to the placenta, while the second, as previously mentioned, ophthalmic and in association with *L. sericata* [117].

*Phormia regina*, distributed in the Holartic region [83], is common in early spring when temperatures are cool and less abundant in summer [107]. The larvae are saprophagous and breed in large numbers on dead bodies [4], thus this species is considered of forensic relevance [82, 135]. Adults of *P. regina* are also attracted by animal feces [136] including those of cats [137]. This fly has been historically reported as the most important secondary species invading wounds in myiasis caused by *Co. hominivorax* [92]. It has been reported as agent of myiasis in cattle [138] and humans, infesting wounds in individuals destituted, neglected or with psychiatric problems [139–142] or causing gastrointestinal [143] and auricular myiasis [140]. Cases of myiasis by *P. regina* were reported in Turkey in the auricular, anal and umbilical regions of three newborn kittens; the multiple site infestation occurred because the kittens had been neglected [144].

*Lucilia eximia*, distributed in the New World [83] is considered a significant indicator of an outdoor forensic scene [145]. In cats, the species has been reported in Brazil as causing abdominal and urogenital myiasis in absence of detectable wounds, but probably attracted by body weakness and skin desquamation [146]. Recently, a similar infestation by *L. eximia* in absence of detectable skin wounds was reported in a domestic dog [147]; probably in this case the predisposing factors were obesity and grooming negligence.

*Lucilia ampullacea*, distributed in the Palaeartic region and India [148], has been reported as agent of myiasis in non-mammal [149], mammal and human hosts [114, 150, 151]. The only reported case of myiasis by *L. ampullacea*, previously mentioned, occurred in Austria and in association with *L. sericata* [114].

*Lucilia caesar* is widely distributed in the Palaeartic region [126]. This species is a “sheep blowfly” in northern Europe [129] and has also been reported as an agent of wound myiasis in humans [152, 153]. There is only one report of myiasis in cats by *L. caesar*, which occurred in Austria [113]. No details were mentioned in this report about the type of myiasis and the number of individuals affected.

*Lucilia coeruleiviridis*, a Nearctic specis, is a very common in southern USA [83]. There is a historical report about an initial myiasis in a cat involving this fly in Virginia (USA) [154]. The host was a kitten in neglected conditions, weak and ill but without wounds; some females of *L. coeruleiviridis* females had laid eggs on its fur [154]. To date, this is the only report involving potential myiasis by *L. coeruleiviridis* in cats.

### Sarcophagidae

This large and widely distributed family includes 3079 [155] larviparous species which can be kleptoparasites, predators, parasitoids, saprophagous and/or agents of myiasis [156]. Some species are forensically relevant [157]. The most important genera of Sarcophagidae causing myiasis are *Wohlfahrtia* Brauer & von Bergenstamm and *Sarcophaga* Meigen (Tables 4, 5). The genus *Wohlfahrtia* includes species causing common obligatory myiasis in warm-blooded vertebrates [158]. The genus *Sarcophaga* includes species responsible for facultative myiasis in vertebrates, but is considered less relevant from a medical and veterinary point of view [158].

Concerning *Wohlfahrtia*, the species reported as agents of myiasis in cats are *Wohlfahrtia magnifica* (Schiner) and *Wohlfahrtia vigil* (Walker). *Wohlfahrtia magnifica* is a Palaearctic species [156] infesting wounds or body openings [6, 105, 159]; it is considered the most relevant agent of obligatory myiasis in Europe, Russia, Asia Minor and North Africa, and frequently parasitizes humans [160]. Only two cases of myiasis by *W. magnifica* have been reported in cats. The first one involved a cutaneous traumatic myiasis in Israel [119]. The second case was reported in Italy and involved a young stray cat; the larvae of *W. magnifica* caused a respiratory tract and oral myiasis with heavy infestation of nose, palate and tongue [161]. *Wohlfahrtia vigil* is common in Nearctic, Palaearctic and Oriental regions [156]. Females usually deposit larvae on the intact skin [4], causing cutaneous furuncular myiasis with lesions containing one to five larvae [6]. Cases of myiasis in cats by *W. vigil* have been reported since 1935 [162] in Ontario (Canada), mostly located on the unfurred regions of the head. Two other cases were reported in Alberta (Canada) [163], one in British Columbia (Canada) [164], and nine in the USA, one in Colorado [67] and other eight cases in Colorado, Kansas, Nebraska and Utah reported by Dr J. T. Alfred (National Veterinary Services Laboratories, USA; 2017, personal communication). In the latter cases the infestations were found on chest, tail, sides, hind legs, abdomen and dorsum, all regions covered by fur (J. T. Alfred, 2017, personal communication); this suggests that larvae of *W. vigil* may reach skin even through the fur. Two other cases of myiasis in cats by *Wohlfahrtia* sp. were reported without identification of the species: the first occurred in Alberta (Canada) and, although unclearly described, apparently was in association with *Sarcophaga* sp. [165], and the second occurred in Colorado (USA) [67].

Only two species of the genus *Sarcophaga*, *S. tibialis* and *Sarcophaga argyrostoma* (Robineau-Desvoidy) have been reported as agent of facultative myiasis in cats [116, 117]. Both species are synanthropic, widely distributed [156] and known since 1913 [128] and 1941 [166], respectively, as agents of myiasis in humans. Moreover, *S. argyrostoma* has been reported in Serbia as an agent of traumatic myiasis in sheep in association with *W. magnifica* [167]. The reported cases of myiasis in cats by these two species occurred recently in Italy and involved wounds respectively caused by a dog bite and a road accident [116, 117]. As previously mentioned, the first case was in association with *L. sericata* [116].

### Muscidae

The only reported case of myiasis in a cat involving a member of the family Muscidae was a respiratory tract myiasis caused by a larva of the cosmopolitan species *Musca domestica* L. (Diptera: Muscidae) [168] in the tracheal lumen (Table 5). After euthanasia, the larva was found inside a nodule in the tracheal epithelium. The authors advanced the hypothesis that the cat became infested by contact with organic matter containing the larvae [168].

## Conclusions

The dipteran species reported as the most frequent agent of obligatory myiasis is *Co. hominivorax*, while that reported as the most frequent agent of facultative myiasis is *L. sericata*, both belonging to the family Calliphoridae. Another six species from this family have been reported as agents of myiasis in cats, thus calliphorids appear prominent as causes of this infestation. Concerning the Oestridae, most cases of myiasis in cats have been reported as caused by *Cuterebra* spp., without a precise identification of the species. The other dipteran families reported as agents of myiasis in cats are the Sarcophagidae and Muscidae. Based on literature data, the factors increasing the risk of myiasis in cats are related to the biology of the agent: when the female lays eggs on grass or wet soil, the cat may be infested while crossing or prowling the infested area, or by ingesting contaminated organic matter. When eggs or larvae are laid directly on the host, the cat may be infested when grooming is not correctly performed, for example when the animal is sick, obese or neglected, or in the absence of parental care. Concerning the anatomical localization of myiasis, the cutaneous type is the most frequent, often connected to open wounds caused by accidents, bites and competitive fighting, or, in old and neglected individuals affected by decubitus ulcers. Other risk factors for urogenital myiasis in females are complications during pregnancy or delivery. Conditions of neglect are a key factor in development of myiasis in stray cats or abandoned kitten. A general survey of myiasis in cats, one of the most common and cherished domestic animals, for the first time gathering widely scattered literature data, could be interesting for veterinarians and animal caretakers. It would be interesting also for entomologists studying the biological and ecological features of Diptera causing this infestation, which could be invalidating and even lethal. Cases of myiasis in cats may appear rare probably because there is also a lack of awareness by cat owners, who underestimate the danger of these infestations. Thus veterinarians should be alerted about the relevance to report these cases, in order to adapt local practices or guidelines to the different distribution of these economically important and often devastating parasites. It would be also useful to extend the survey on myiasis to other household pets, for comparative and epidemiological studies. The literature data presented in this review could also be useful for the development of guidelines and to promote a fruitful collaboration among veterinarians, entomologists and researchers interested in the field.

## Data Availability

The data supporting the results and conclusions of this article are included within the article. The raw datasets are available from the corresponding author upon reasonable request.
